# Public legitimacy of healthcare resource allocation committees: lessons learned from assessing an Israeli case study

**DOI:** 10.1186/s12913-022-07992-6

**Published:** 2022-06-02

**Authors:** Yael Assor, Dan Greenberg

**Affiliations:** 1 Department of Anthropology, University of California, Los Angeles, USA; 2 Edmond J. Safra Center for Ethics, Tel-Aviv University, Tel-Aviv, Israel; 3 Department of Health Policy and Management, School of Public Health, Faculty of Health Sciences, and the Guilford-Glazer Faculty of Business and Management, Ben-Gurion University of the Negev, Be’er-Sheva, Israel

**Keywords:** Health Policy, Accountability for Reasonableness, Legitimacy, Israel

## Abstract

**Background:**

The National Health Insurance Law enacted in 1995 stipulates a list of health services to which all Israeli residents are entitled. For the past 20 years, the list has been updated annually, as a function of a predetermined budget, according to recommendations from the Public National Advisory Committee (PNAC), which evaluates and prioritizes candidate technologies. We assessed the legitimacy of this resource-allocation process as reflected in Israeli public discourse and its congruence with the accountability for reasonableness (A4R) framework.

**Methods:**

A qualitative analysis of public discourse documents (articles in the print media, court rulings and parliamentary debates (*N* = 119) was conducted to assess the perceived legitimacy by the Israeli public of the PNAC. Further content analysis of these documents and semi-structured interviews with stakeholders (*N* = 70) revealed the mainstays and threats to its legitimacy. Based on these data sources, on governmental documents specifying PNAC's procedures, and on data from participant observations, we assessed its congruence with A4R’s four conditions: publicity, relevance, revision and appeals, regulation.

**Results:**

The PNAC enjoys ongoing support for its legitimacy in Israeli public discourse, which stem from its perceived professional focus and transparency. These strengths are consistent with the A4R’s emphasis on the publicity and the relevance conditions. The three major threats to PNAC's legitimacy pertain to: (1) the composition of the committee; (2) its operating procedures; (3) its guiding principles. These perceived shortcomings are also consistent with incongruencies between PNAC's work model and A4R. These findings thus further support the empirical validity of the A4R.

**Conclusion:**

The analysis of the fit between the PNAC and A4R points to refinements in all four conditions that could make the A4R a more precise evaluative framework. Concurrently, it highlights areas that the PNAC should improve to increase its legitimacy, such as incorporating cost-effectiveness analyses and including patient representatives in the decision-making process.

Hebrew and Arabic abstracts for this article are available as an additional file.

**Supplementary Information:**

The online version contains supplementary material available at 10.1186/s12913-022-07992-6.

## Introduction

The healthcare system in Israel operates under the National Health Insurance Law (NHIL) enacted in 1995. Publicly funded healthcare is provided by four competing non-profit health plans (Health Maintenance Organizations, HMOs). The NHIL stipulates a minimum National List of Health of Services (NLHS or benefits package) that the health plans must provide to their members. Throughout the past 20 years, Israel has deployed a unique annual resource allocation mechanism to determine which new medical technologies will be covered and reimbursed by the NLHS, carried out by the Public National Advisory Committee (PNAC).

The process of updating the NLHS has been described and examined in more detail in previous studies [1–3]. Every year, hundreds of applications for technologies to be included in the NLHS are submitted by various stakeholders. As the budget allocated for this update is limited, most of these technologies, some of which have substantial value for patients, are denied coverage. In this scenario where so many people’s health needs are unmet, it is particularly challenging to establish and maintain the legitimacy of the prioritization process. The current study considers findings concerning the legitimacy of this prioritization process and its causes in order to examine the applicability of Accountability for Reasonableness (A4R), the leading framework for bioethical legitimacy [4].

This paper has a three-tiered structure. The first tier presents the process of updating the NLHS in Israel, as well as the A4R framework. The second tier discusses the findings of a study examining the legitimacy of the process as reflected in public discourse, defined here as media, parliament and court deliberations and rulings. We analyze the major contributors to its legitimacy as articulated in the media and in interviews with stakeholders involved in the prioritization process, including patients, journalists, and committee members. These elements are shown to correspond closely to the A4R conditions, thus providing empirical support for the validity of this theoretical framework.

In the third tier, we assess the overall compatibility of the updating process with A4R’s four conditions. This assessment follows up on a previous assessment of the PNAC’s congruence with A4R conditions conducted a decade ago [1]. In the ten years since then, certain changes have been made in the process, primarily an increase in efforts toward greater public transparency, but overall, its structure has not undergone a radical change. This follow-up thus highlights the changes made to the PNAC model in the last decade.

### Legitimacy and the Accountability for Reasonableness (A4R) framework

Drawing mainly on political thought and organizational theory, legitimacy pertains to “the perceived appropriateness of an organization to a social system in terms of rules, values, norms, and definitions.” [5] Legitimacy, then, is a quality attributed to a body by its constituents [6]. In fact, as Suchman [7] noted, this quality encompasses three different dimensions: pragmatic, moral, and cognitive. Pragmatic legitimacy is the extent to which the organization serves constituents’ interests and meets their expectations. Moral legitimacy is the consistency between the organization and its constituents’ values and beliefs. Cognitive legitimacy is the extent to which constituents consider the organization’s activity as understandable.

Evaluations of an organization’s legitimacy occur at the collective and the individual level. Legitimacy at the collective level concerns validity, glossed as “the extent to which there appears to be a general consensus within a collectivity that the entity is appropriate for its social context.” [8] At the individual level, legitimacy concerns propriety: an evaluator’s approval of the organization, its actions, or its practices as desirable and appropriate [9, 10]. Studies have shown that collective validity evaluations significantly affect individuals’ propriety evaluations, with particular influence to validity evaluations expressed by the media, government institutions, and the judicial system [11].

Studies about organizational legitimacy centrally focus on ways to increase validity evaluations [12]. In bioethics, a particularly helpful tool to assess a body’s legitimacy and how it could be increased is the A4R framework. A4R is based on the notion that as long as a decision-making procedure is considered fair, its results will be broadly accepted, even if they are contentious. The A4R defines four conditions for fair resource allocation processes, each building on the fulfilment of its predecessor. The first is the publicity condition, where the reasons for the allocation decisions (like the decisions themselves) must be made publicly accessible. The second is the relevance condition, which states that the rationales for the decision-making process must be considered reasonable explanations for how they were made. It assumes that a rationale will be viewed as reasonable “if it appeals to evidence, reasons, and principles that are accepted as relevant by fair-minded people.” The third, the revision and appeals condition, specifies that there should be mechanisms to challenge these decisions. The fourth, the regulative condition, states that there should be some kind of public regulation to ensure that the other three conditions are met [4].

In the twenty years that have passed since its publication, the A4R has become the predominant evaluative mechanism for healthcare resource-allocation endeavors worldwide [13, 14]. Broadly speaking, there are two main types of evaluation. One views the A4R as an a-priori evaluative standard. These evaluations focus on the fit of a resource allocation process to the A4R model, and suggest ways to improve it based on the A4R [1, 15–18]. An example of this type of evaluation is the 2010 assessment of the PNAC’s legitimacy, which specified areas the committee needed to improve to better meet the A4R conditions [1].

The second orientation takes a critical approach to A4R as an evaluative standard. While these studies also evaluate the fit between the A4R and a resource allocation process, here the focus is on areas where A4R could be improved or point to other elements that should be taken into consideration in addition to the A4R [19–21]. For instance, a study of healthcare allocation decisions in sub-Saharan Africa argued that supplementing the A4R with evaluations of adherence to a communal responsibility principle would provide a more comprehensive evaluation of public legitimacy in this setting [20].

Importantly, some of these studies also draw on the A4R to suggest improvements in the decision-making process itself. We follow this lead and take a bi-directional approach. On the one hand, we provide a critical examination of the A4R conditions and suggest how they could be refined to better assess legitimacy. On the other, we base our evaluation of the fit of the PNAC to the A4R and list areas that would improve its functioning. This bi-directional approach is grounded in an empirical analysis of the PNAC’s legitimacy as expressed in public discourse. These findings provided us with data to examine the empirical validity of the A4R and determine the factors leading to a more robust evaluative framework.

### The process of updating the national list of health services

Israel’s NHIL enacted in 1995 stipulates the NLHS that the four health plans operating in Israel must provide to their members. Since 1998, the Israeli government has allocated an annual budgetary extension earmarked for the inclusion of new technologies on this list. The process of updating the NLHS starts every year with a call for applications of new technologies or new indications for technologies already covered in the list. Applications for technologies must include a detailed dossier and can be submitted by various stakeholders including, but not limited to, pharmaceutical and medical device companies, health plans, professional medical societies, physicians and the general public. In the first round of the assessment process, the technology forum at the Ministry of Health (MoH) reviews each application and determines whether the technology should be considered for further assessment. For example, further assessment is not needed when a technology is already included in the NLHS for the same indication.

The next phase includes a health technology assessment (HTA) conducted by the MoH for each eligible technology. The assessment includes considerations related to the technology’s regulatory approval status, safety, efficacy, epidemiological data, expert physicians’ reviews and its rankings by relevant medical societies in Israel, existing experience with the technology in Israel and in other countries, and a comparison to alternatives.

Towards the last quarter of the year, the Ministers of Health and Finance appoint a public national advisory committee (PNAC) responsible for the ranking and prioritization of the candidate technologies. The committee is composed of 18–20 members of whom about half are MoH, Ministry of Finance and health plan representatives, and the other half are expert physicians and public representatives (usually an ethicist, a representative of the Jewish clergy, and directors of non-medical nonprofits). As discussed below, despite some calls for change, the committee does not include any patient advocate representatives.

The committee’s discussions contain two phases. In the first, each technology is ranked independently of the other technologies. Only technologies attributed the three highest rankings, A8 to A10, continue on to the next phase, where they are all considered together while evaluating their budgetary impact and other factors.

Throughout the PNAC’s deliberations, a sub-committee including representatives from the Ministries of Health and Finance and the four health plans convenes to determine the total estimated budget needed to cover each technology, in terms of the number of Israeli patients likely to use it and the incremental cost per patient for each technology with the highest ranking. In the absence of national patient registries that would provide information about Israel’s patient population, the number of patients in need is assessed using data provided by the health plans, medical societies, expert clinicians and other sources.

By the end of the calendar year, the PNAC convenes for several sessions to make its recommendations as to which technologies will be covered and reimbursed and for which populations and indications. The recommendation can apply to the entire target patient population or be for restricted use. For example, a technology can be approved solely as an advanced treatment line after the failure of previous interventions. The decisions are usually made by consensus. Although not all the preparatory work is documented in transcripts or journalistic reporting, since 2010, the PNAC transcribes its sessions and makes them available to journalists. However, as discussed below, this move toward greater transparency still has its limitations.

## Methods

### Data collection

Data were collected by the first author from two sources (see Table 1): (1) Semi-structured interviews with all parties involved in the work of the PNAC, including former and acting committee members and staff, journalists, representatives of patient advocates, and representatives of pharmaceutical companies (*n* = 70). (2) Media, parliament, and court documents on the PNAC (*n* = 119). All these documents are accessible to the public and available on the internet. These data sources were selected as they reflect the main avenues for evaluations of legitimacy at the collective level [11].

**Table 1:** Types of data collected

Dataset	Subdivision	Frequency
Interviews	Journalists	8
	Patient organization representatives and health activists	11
	Politicians addressing PNAC’s work in parliament	4
	PNAC administrative staff	8
	PNAC committee members	30
	Lobbyists	3
	Representatives of pharmaceutical companies	2
	Academic experts in contact with the PNAC	4
	Total	70
Public Discourse Documents	Media op-eds/interpretations/investigative stories	110
	Supreme Court rulings	3
	Parliamentary debates	6
	Total	119

Interviews were conducted from 2015 to 2018, under ethical approval of the Institutional Review Board of the University of California, Los Angeles. Interviewee’s sampling occurred through a snowball method. Most interviews lasted about 1.5 hours, and included questions on the respondent’s impressions of the committee’s functioning and work procedures, its level of public legitimacy, and its sources of legitimacy (if assessed to be publicly legitimate). The interviews took place at the interviewees’ offices, homes, or in coffee shops. All interviewees signed informed consent forms, and all the interviews were recorded and transcribed by the first author.

All the documents surveyed were published between 2010 and 2020. They include six Israeli parliament (Knesset) debates and three court rulings on the PNAC, and 110 articles appearing in the press presenting positions on the PNAC, including interviews with people involved in its work (as members or stakeholders), interpretive reporting essays, and investigative stories. The corpus did not include media coverage that simply reported on ongoing discussions or resolutions of the PNAC. Although a committee resolution report could contain interpretive comments, the majority did not contain any such statements, so that the differentiation between these reports and documents manifesting explicit stances on the committee’s work was mostly straightforward.

To evaluate the fit of the PNAC with A4R, these two data sources were used along with two additional sources: (1) Governmental documents relating to the PNAC’s work; i.e., the MoH protocol of PNAC procedures [22], the NHIL, PNAC session transcriptions, and the health technology assessment template; (2) Participant observations in sessions open to the public concerning the PNAC’s work, approved by the same Institutional Review Board as were the interviews. The first author conducted these observations as part of her anthropological research on the causes of the PNAC’s public legitimacy.

### Data analysis

Data analysis for this study was conducted according to the “grounded theory” methodology [23] and using MAXQDA software. In order to catch both explicit and implicit articulations concerning PNAC’s legitimacy and its causes, the first author conducted a manual coding process, in which she reviewed all the data in a recursive process, returning to code all the previous data each time she added a new code. To analyze the PNAC’s legitimacy as expressed in public discourse, the first author reviewed and coded any explicit references to its overall legitimacy or lack of legitimacy and any references to the public’s trust/mistrust in its workings. Based on results suggesting its overall strong public legitimacy (see below), articulations expressing the causes for its legitimacy were coded in the public discourse documents and the interview transcripts.

For the public discourse documents, all articulations specifying why the PNAC is legitimate or trustworthy were coded, as were statements questioning its legitimacy. For the interview transcripts, any responses to direct interview questions on this topic were coded and then collated into major themes. Subsequently, a second analysis was conducted to code any unsolicited articulations of these themes.

Analysis of the PNAC’s congruence with the A4R framework was carried out jointly by the first and second author. In the analysis, we considered the fit between the A4R conditions and the PNAC procedures. Since the government documents on the PNAC procedures are outdated in part, wherever there was a discrepancy between them and the other data sources, the latter were preferred. For instance, according to the 2010 protocol, the PNAC’s initial round of technology ranking is conducted by an internal MoH team. However, all the other data sources showed that PNAC stopped doing this shortly after the protocol was published. In the last 10 years, this ranking has become the first phase of the PNAC’s deliberation process.

Furthermore, in our analysis we examined changes in PNAC procedures over time, whether short-lived or not. Information on these modifications came mainly from interviews and from documents announcing them, such as a call to include patients’ perspectives in the data presented to the committee. We also drew on the second author’s first-hand experiences, since he has taken an active role in two short-lived initiatives: incorporation of a cost-effectiveness analysis into the PNAC’s decision-making process, and the incorporation of patients’ perspectives in the data presented to the PNAC for deliberation.

## Results

### PNAC’s level of legitimacy and its causes

The PNAC was viewed as legitimate in all the public discourse documents in the corpus. Out of 110 media documents analyzed, the majority (*n* = 68) included statements supporting its legitimacy. For instance, an op-ed by a prominent patients’ rights advocate stated that the PNAC is “the only body that is professionally and publicly authorized to determine what should be included or excluded from the [health services] basket.” [24]

Only a few documents disputed the PNAC’s legitimacy (*n* = 4). All four argued that its decisions should be made by the health plans separately according to the makeup of its members. A considerable number of documents did not contain any statements concerning its legitimacy/non-legitimacy. All the court and parliamentary documents included statements supporting the PNAC’s legitimacy. For example, in a ruling concerning a patient organization’s appeal for an accelerated deliberation process on their medication, one of the ways to bypass the PNAC process, stated: “The committee must be allowed to complete its deliberations and we see no reason to interfere in its professional process.” [25]

Subsequent analysis of the public discourse documents and interview datasets revealed three major themes that contribute to the PNAC’s legitimacy. Interestingly, these themes correspond with the three dimensions of legitimacy Suchman discerns [7]:

1. PNAC’s professional focus and perceived objectivity. Evaluations of PNAC’s legitimacy in public discourse documents predominantly related to legitimacy’s moral dimension, stressing “objectivity” and “professional focus” as core ethical values that should guide its work [3]. In most of these documents, it is further argued that PNAC indeed follows these values. This notion appeared in all parliamentary and court deliberations, and was particularly prominent in media articles and in most interviews. As argued in one of the major profile articles about the PNAC’s work, “it is a rare island within Israeli public administration which is not governed by pressure and interests … Apparently it is possible to act professionally and impartially even when faced with budget limitations and pressure from stakeholders.” [26] In this article and most other sources addressing PNAC in a similar manner, this reference to its professional focus pertained to the fact that it is not a committee composed of politicians but rather made up of professional experts, and the fact that the PNAC’s deliberations are based on HTA, which many interviewees viewed as guaranteeing an “objective” decision-making process.
2. The PNAC’s relative transparency. Corresponding with the cognitive legitimacy dimension, the second most prominent theme pertained to PNAC’s efforts to make its decision-making process understandable to the public by enacting some transparency measures. This notion was discussed primarily by the interviewees and in the media but not in parliamentary or court deliberations. References to the committee’s transparency mainly pertained to the publication of the transcript of its discussion sessions and journalists’ reports from committee sessions. In fact, as some interviewees noted, the crucial turning point in increasing its public legitimacy was the PNAC’s 2010 decision to open its sessions to media coverage and release its deliberation transcripts. As one journalist put it in an interview, “[U] p to then, we simply did not know what was happening there, so we imagined the worst. When they allowed us in, we realized it was actually a pretty boring, straightforward process, and we informed our readers accordingly.” Nonetheless, as discussed below, the belated publication of committee transcripts remains a central challenge to increasing its legitimacy.
3. The PNAC’s willingness to discuss cutting-edge technologies and include a comprehensive list that covers a variety of health conditions. Consonant with the dimension of pragmatic legitimacy, this theme relates to the public interest in including innovative technologies that appeal to the needs of as many constituents as possible. This notion was highlighted in particular in the media but did not appear in parliamentary or court deliberations and scarcely appeared in the interviews.

Although most of the public discourse documents and the interviewees overall supported PNAC’s legitimacy, some questioned certain facets of its operation. The three prime issues were as follows:

1. The composition of the PNAC. Discussed in approximately one-third of the media articles and the interviews, this issue was approached from two points of view. Primarily this pertained to the lack of representation of patients’ organizations. As a former committee member noted in an interview, “[E] xcluding patient representatives from participation is not just patronizing, but also foolish in the sense that it would have garnered further support for the committee as truly representative of the Israeli public.” Another issue related to PNAC membership, which came up mainly in interviews with former and acting committee members and staff, as well as in a few media articles, was the presumed power imbalance between the health plan representatives on the committee and other members. While the former are appointed for years, and are thus experienced PNAC members, the latter can change yearly and thus do not have as much knowledge of the committee’s workings.
2. PNAC’s work procedures. This point came up in many of the media articles and in approximately half of the interviews. Two main issues were cited. The first, which was seen as the most crucial, was the PNAC’s transparency model. Alongside acknowledgment of its efforts to achieve transparency as a leading cause of its legitimacy, many noted shortcomings in its transparency model. These are discussed in detail in the next section. The second issue was PNAC’s decision model, which prohibits changes in decisions once they have been officially announced (see below).
3. PNAC’s decision-making principles. A point that came up in a quarter of the media articles and in the interviews concerned what guides PNAC’s decisions. This related to PNAC’s lack of consideration of the cost-effectiveness of technologies. Some of the interviewees also noted the lack of guidelines related to specification and prioritization, as elaborated below [22].

Overall, the findings thus pointed to two main reasons for the PNAC’s relatively high level of public legitimacy: its perceived professional focus and objectivity, and its transparency. These two features correspond to the A4R’s two main conditions of publicity and relevance, suggesting that the A4R is empirically appropriate for assessing legitimacy. Three major challenges to PNAC’s legitimacy were also addressed: the composition of its membership, its work procedures, and its decision-making principles. An analysis of the ways in which these challenges map onto the A4R framework makes it possible to investigate exactly where and why the factors supporting and potentially undermining the PNAC’s legitimacy come into play.

### Assessment according to A4R

#### The publicity condition

The PNAC meets this condition partially, although there has been improvement from a decade ago. The PNAC publishes a summary of its decisions on the MoH website, and in a broadly-covered media announcement. However, publicity of PNAC’s rationales for reaching their decisions is only partial. While some steps have been taken towards increasing its transparency over the last ten years, this remains far from full transparency (see Table 2).

**Table 2:** PNAC Fulfilment of the Publicity condition

Condition(s)	Actual fulfilment	1999	2010	2019
Decisions are publicly accessible	Summary of the committee’s decisions are posted on the MoH website	No	Yes	Yes
Rationales are publicly accessible	The public can follow the decision-making process as it happens or shortly afterwards	No	Partly	Partly
	The public can follow the decision-making process through published transcripts	No	No	Yes
	The public can follow all steps of the decision-making process	No	No	No

In 2009, following a decade of searing accusations in the media concerning the PNAC’s vested interests and decisions, the PNAC changed its dissemination policy and started to make its decision-making process more open to the general public. Summaries of the main PNAC decisions are posted on the MoH website, several days after each committee session. It also publishes transcripts of the committee’s discussion sessions on MoH’s website, and allows health journalists to attend its sessions. However, this is not a model of unrestrained transparency in which the public can follow the deliberation process in an unfiltered manner and in real time. Session transcripts, which should be published up to 60 days after the committee submits its recommendations, are typically published 3–7 months after the final PNAC recommendations are made public. Moreover, although journalists are allowed to file general reports on the sessions, they are not allowed to make its final decisions public before the PNAC formally submits them. No committee member is quoted by name in either the transcripts or the media reports. These transcripts can also be censored to some extent in cases where the deliberation process touches upon particularly volatile content, such as the country’s role in funding technologies for predicaments caused by patients’ engagement in health risk behaviors.

The PNAC’s rationale behind these limitations is to avoid pressure on the committee or specific members while they are engaged in the deliberation process. The PNAC also uses this argument to justify its longstanding refusal to make its subcommittee’s rationales for deciding on patient numbers, pricing and each technology’s estimated budget impact public. Thus, one of the major determinants of the PNAC’s allocative decisions remains completely shielded from public scrutiny. Finally, deliberations held in the MoH’s technology forum concerning which technologies are eligible to be submitted to the PNAC for consideration are kept confidential.

#### Relevance condition

This condition can be divided into two components: (1) relevant considerations for decision-making, and (2) broad representation in the decision-making body that together reaches mutual terms of cooperation. Neither are fully met (see Table 3).

**Table 3:** PNAC fulfilment of the Relevance Condition

Condition(s)	Actual fulfillment	1999	2010	2019
Considerations of decision-making are accepted as relevant by designated fair-minded people	Evidence accepted as relevant	Yes	Yes	Yes
	Reasons accepted as relevant	Yes	Yes	Yes
	Principles accepted as relevant	No	No	No
“Fair-minded people” are designated with finding terms of cooperation	Representation of all relevant stakeholders and weights given to societal and patient preferences	No	Partly	Partly

As the relevance condition suggests, the considerations for decision-making include evidence, reasons, and principles. The evidence the PNAC uses to make its decisions are each technology’s HTA, which the interviewees and public discourse documents considered to be high-quality and objective [3].

The reasons for each decision are not fully disclosed to the public, as discussed in the publicity condition section. However, since the PNAC applies a consensus-based decision model, it could be claimed that its decisions are based on reasons that the committee members find to be overall acceptable, even if at times this involves some negotiation and/or persuasion.

The PNAC’s guiding principles depart from the A4R in several ways. Although the MoH protocol lists several principles guiding the PNAC’s decisions [22], these principles remain ambiguous. The protocol does not prioritize these principles, so that it remains unclear which principle is more crucial in cases of conflict. In practice, the PNAC often encounters such conflicts, for instance between technologies improving patients’ quality of life as compared to technologies deemed to be “life-saving”, two principles noted in the protocol. It is perhaps not surprising that only one committee member out of 30 interviewed noted that these principles informed his decision-making.

Another key discrepancy between the PNAC and A4R concerns the latter’s specification that decisions should maximize health outcomes and provide “good value for money.” Over the course of one year, from 2009 to 2010, the MoH tried to incorporate a cost-effectiveness analysis into its HTA. However, it did not follow through on this initiative for lack of manpower. Since then, PNAC has not formally implemented value-for-money concerns in its deliberations, although it is one of the main considerations in similar processes worldwide.

The membership composition of the PNAC also fails to fully meet the A4R’s relevance condition. Despite various attempts to address this issue, the PNAC still does not represent all stakeholders, since patient representatives are not members of the committee. Although the PNAC does include several “public representatives,” interviews with them indicated that they do not see themselves as representatives of patients’ interests. Rather, almost half, who are mostly clergy and ethicists, did not see themselves as representatives of the Israeli public at large but instead as contributing their professional viewpoints.

The 2009 assessment of PNAC’s adherence to the A4R described a short-timed model to incorporate patients’ views termed the “Health Parliament”.^1,^ [27] In 2010, around the time that the committee was taken to court for its lack of patient representation [28], the committee initiated public hearings in which patients could present their positions to the committee. This model was discontinued two years later, with a change in committee membership.The court appeal also did not carry news concerning patients' participation in the committee, as the appeal was declined. The court argued that there was no need to enforce patients' participation since they were represented on the Health Council, whose role is instituted in Israel’s NHIL and who should be convened to confirm the PNAC’s decisions. In practice, however, the Health Council automatically approves the PNAC’s decisions without convening, thus denying patients their one chance at representation in this decision-making process.

In 2019, the PNAC launched a third attempt to incorporate patients’ voices by soliciting patients’ written testimonials through an online forum, which are then presented to the committee during its deliberations. This effort is still ongoing. These three attempts suggest some PNAC acknowledgment of the difficulty involved when excluding patients’ involvement in the decision-making process. However, it also attests to the committee’s continued reluctance to including patient representatives as full-fledged members. The main justification for this refusal that committee members voiced in interviews was their concern that these representatives would be biased to include the treatment of the medical conditions most closely connected with their own afflictions. While this is a plausible argument, it is worth inquiring why this argument has not been used against the expert physicians on the committee, who are likely to be biased in favor of their own fields of expertise. Moreover, in Israel there in an umbrella organization that represents patient groups in front of the MoH and other governmental bodies called the Organization for Patients’ Rights. Including a representative from this organization could ensure the minimization of individual cases of bias.

#### Revision and appeals condition

Out of A4R’s four conditions, this is the one the PNAC fulfills the least, although there has been some improvement from a decade ago (see Table 4). In the past decade, the PNAC has instituted a timeframe for stakeholders to file appeals between the two phases of its deliberation process. After the publication of the technology rankings on the MoH website, stakeholders can send a letter of appeal asking the PNAC to reopen a given technology’s ranking deliberations. All appeals are collated and discussed in a special “appeals session” the committee holds before proceeding to the second phase of deliberation. Importantly, no such process occurs when the PNAC submits its final recommendations, making the in-deliberation appeals a unique opportunity for stakeholders to challenge its determinations. Technologies that are denied coverage can be re-submitted for inclusion in the NLHS the following year.

**Table 4:** Fulfilment of the revision and appeals condition

Condition(s)	Actual fulfillment	1999	2010	2019
Dispute resolution mechanism	Appeals related to the priority and ranking of technologies during committee deliberations	No	No	Partly
	Appeals of final recommendations	No	No	No
Opportunities for revision and improvement of policies	Procedure to de-list technologies already included in the benefits package	No	No	No
	Procedure to adjust funding for technologies with an FDA “accelerated approval” status	No	No	No

In terms of opportunities to revise and improve policies, although Israeli legislation mentions a procedure for de-listing technologies from the NLHS, this procedure is not utilized. The major hurdle is that de-enlistment must be decided in a parliamentary committee. Their debates can be significantly influenced by stakeholders with various interests, such as (but not limited to) the pharmaceutical company whose technology is threatened with removal. Thus, even technologies which have been shown to be clearly ineffective or unused are never removed from the list.

#### Regulative condition

There have not been any changes in PNAC’s adherence to this condition in the last ten years (see Table 5). As has been the case since its inception, the PNAC is not obligated by any statutory clause to make its decisions or rationales public. Instead, the PNAC’s workings are outlined in the 2010 MoH protocol that serves as a binding reference in appeals submitted to the Israeli Supreme Court. The procedure for de-listing technologies is instituted in Israel’s NHIL. The committee’s appeals-during-discussion procedure is mentioned in the 2010 protocol.

**Table 5:** Fulfilment of the regulative condition

Condition(s)	Actual fulfillment	1999	2010	2019
Public regulations for failure to meet previous conditions	Public regulation concerning publicity	No	No	No
	Public regulations concerning relevance	No	Partly	Partly
	Public regulations concerning revisions and appeals	Partly	Partly	Partly

## Discussion

As the results from the public discourse analysis suggest, the PNAC enjoys stable and ongoing support for its legitimacy to make difficult resource allocation decisions. The two main reasons for its legitimacy are its professional focus and objectivity, and its transparency, reflecting the moral and cognitive dimensions of legitimacy, respectively. Both issues are covered directly in the A4R framework. The PNAC’s reliance on health assessments and professional membership, the two factors included in under “professional focus and objectivity,” correspond to the A4R’s relevance condition. The PNAC’s transparency corresponds directly with the A4R’s publicity condition. The findings here thus confirm A4R’s emphasis on the publicity condition and the relevance condition as the two most important conditions for ensuring legitimacy.

Relatedly, the main challenges to the PNAC’s legitimacy articulated in public discourse and in interviews were the same as the major incongruences with A4R. The lack of patient representation and the lack of consideration of cost-effectiveness data correspond to the A4R’s relevance condition. Issues concerning the PNAC’s work procedures correspond to limitations on its transparency, such as the lack of subcommittee meeting transcripts, are a match with the A4R’s publicity condition. The lack of procedures to revise and adjust the PNAC’s decisions correspond to A4R’s revision and appeals condition.

Thus overall, the factors contributing and detracting from the PNAC’s legitimacy correspond to its relative fit with A4R, suggesting empirical support for the A4R’s validity. The examination of the fit between PNAC and A4R elicits two sets of implications of this study. The first set concerns some refinements that could make A4R a more precise evaluative framework. The second set of implications concerns the areas the PNAC should improve to increase its legitimacy.

### Implications I: refinements for A4R

The PNAC’s belated publicity of its transcripts underscores that publicity is not merely about making considerations public, but also that doing so must occur in a timely manner. That is, publicity is worthwhile only if the topic remains relevant. One example is the FDA’s decision to conduct its deliberations concerning the COVID-19 vaccine on live broadcasts. Had the FDA released discussion transcripts several months after submitting its conclusions, it would not have been able to so quickly dispel criticism as to its deliberation process.

The controversy over the PNAC’s refusal to allow patient organizations to serve as committee members is also related to one of the critiques of the A4R’s relevance condition. The A4R condition states that decisions should be made by “fair minded people.” However, critics have argued that this is a particularly ambiguous statement since it does not indicate who should determine which individuals qualify as “fair minded.” [29–31] This point emerged clearly in the case of the PNAC. In interviews with former and acting PNAC members, the dominant argument against incorporating patient organizations’ representatives was that they would be biased. However, the court case dealing with this issue accepted the general argument that patients should be represented in the decision-making process, and only ruled against the appeal because it thought that there was already representation through the Health Council. This suggests that the PNAC and the Israeli court have different views as to whether patients are “fair minded” people. To increase the impact and validity of A4R, it might be worthwhile to specify who should determine which individuals are “fair minded”: is it up to the resource allocation mechanism or to other social institutions, such as the court?

The PNAC’s incomplete fulfilment of the revision and appeals condition makes it clear that it is not enough to institute dispute resolution mechanisms and provide opportunities to revise decisions. Rather, there should be reference to the actual feasibility of these measures. In the PNAC’s case, Israeli legislation formally includes references to all facets of the revision and appeals condition. However, the politically-dependent procedure for revising decisions places a stranglehold on its implementation. A more robust wording of the revision and appeals condition would better address the feasibility of these mechanisms and procedures.

Finally, the PNAC case suggests there are potential benefits in specifying what is meant by “regulation of the process” in the A4R’s regulative condition. With respect to the PNAC, the MoH does not follow some of the work procedures listed in the NHIL or the 2010 PNAC protocol. So far, there has been no enforcement of existing regulations, voluntary or public. In this regard, A4R’s regulative condition might be refined to address regulations’ enforcement.

### Implications II: amelioration of the Israeli PNAC

To fulfil the publicity condition, PNAC should ensure the timely publication of its discussion transcripts. In addition, the PNAC should apply the same publicity standards used for its committee discussions to its subcommittee procedures and the MoH preliminary technology forum. Publishing the subcommittee’s and technology forum’s deliberations would also contribute to better compliance with A4R’s relevance condition. Without such publications, there is no way to determine the extent to which the general public finds the PNAC’s reasons for resource allocation acceptable.

The PNAC’s principles for decision-making should also be improved. Principles should be prioritized so that it is clear which are more crucial in case several principles are invoked. Methods such as multi-criteria decision (MCDA) analysis that assign weights to each principle could be considered [13]. In addition, the PNAC should incorporate a “value for money” deliberation principle. To do so, it must re-incorporate a cost-effectiveness analysis in its HTA.

To further satisfy the relevance condition, the controversy concerning patient representatives’ membership on the PNAC should be resolved. The key is identifying who determines which individuals are qualified as “fair minded”. We believe that as is the case in other disputes concerning governmental decision-making procedures, the Israeli court should have the final word.

The PNAC’s appeals and revisions process should also be improved. The most urgent issue is the fact that the Health Council is not convened frequently and therefore does deliberate appeals against PNAC’s final decisions. We realize that this process might jeopardize the prompt deployment of PNAC’s resolutions and thus potentially hurt patients waiting eagerly for the public financing of technologies. However, this is the appeals mechanism instituted by law, and therefore unless there is an alternative legislation or procedure, it should be adhered to.

One step that could help facilitate the adoption of these measures would be to pass a PNAC law. As the A4R’s regulative condition argues, enshrining the resource allocation body in a parliamentary decision would guarantee its ability to adhere to fully all conditions.

### Limitations and future research

This study’s primary limitation and potential for future research relates to the offered refinements to the A4R framework. While the data on PNAC’s evaluated legitimacy allows a unique viewpoint to assess A4R’s utility, drawing on one case study limits the presumed validity of the offered refinements. Therefore, further research should deploy the analysis process conducted in this study on other healthcare resource allocation bodies, in Israel and beyond.

At the theoretical level, future research should expand the dialogue between studies of organizational legitimacy and studies about the A4R framework. One particular field that might be generative is considering how strategies for increasing organizational legitimacy correspond with the measures offered by the A4R framework, and vice-versa. Putting into conversation these two literatures might reveal potential blind spots in both fields.

Further limitations of this study pertain to its manner of assessing PNAC’s legitimacy. This study focused on the macro level of collective legitimacy evaluations as expressed in public media documents. It therefore does not provide information about individuals’ evaluations of PNAC’s legitimacy. Additionally, due to unavailability of earlier data, this study only relates to collective evaluations of legitimacy in PNAC’s second decade (2010–2020). Public discourse documents from PNAC’s first decade (2000–2010) would have allowed to trace any trends in its legitimacy evaluations since its foundation.

## Conclusion

The process of updating the NLHS in Israel enjoys a relatively high level of public legitimacy. However, the allocated budget is not adjusted proportionally despite the rapid advances in medicine and the rising costs of technologies, which combine to challenge its legitimacy. Ensuring greater congruence between PNAC’s mode of operation and A4R can help to achieve greater legitimacy. At the same time, A4R itself would benefit from proposing a more robust framework by adopting some refinements to its four conditions.

## Supplementary Information

**Additional File 1.** Hebrew and Arabic abstracts.

## Data Availability

All data generated or analyzed during this study are included in this published article.

## Abbreviations

A4R: Accountability for Reasonableness
NLHS: National List of Health of Services
PNAC: Public National Advisory Committee
NHIL: National Health Insurance Law
MoH: Ministry of Health
HTA: Health technology assessment
