# Supplementary material for: Public legitimacy of healthcare resource allocation committees: lessons learned from assessing an Israeli case study
Source: BMC Health Serv Res. 2022 Jun 2;22:737. doi: 10.1186/s12913-022-07992-6 (PMC9161764; doi:10.1186/s12913-022-07992-6)
Supplement: Supplementary file 1 — Additional File 1. Hebrew and Arabic abstracts. [file 12913_2022_7992_MOESM1_ESM.pdf]

תקציר:

רקע:

חוק ביטוח בריאות ממלכתי, שנחקק בשנת 1995, מפרט את רשימת שירותי הבריאות שכל תושב ותושבת ישראלים זכאים להם. בעשרים השנים האחרונות הממשלה מקצה תקציב שנתי ייעודי לעדכון רשימה זו. עדכון הרשימה נעשה על פי המלצותיה של הועדה הציבורית להרחבת סל שירותי הבריאות, הידועה בציבור כ"ועדת הסל", אשר מעריכה ומתעדפת את כל הטכנולוגיות המועמדות. במאמר זה אנו בוחנים את הלגיטימציה של הליך הקצאת המשאבים הזה כפי שהוא משתקף מהשיח הציבורי בישראל. כמו כן, אנו בוחנים את התאמת אופן עבודתה של ועדת הסל למודל הביואתי accountability for reasonableness (בקיצור, A4R).

שיטות:

הערכת הלגיטימציה שניתנת לוועדת הסל בציבור הישראלי מתבססת על ניתוח מסמכי שיח ציבורי (מאמרים מהעיתונות המודפסת, פסיקות בית משפט, דיונים בכנסת, N=119). ניתוח תוכן של מסמכים אלו ושל ראיונות חצי מובנים עם בעלי עניין מרכזיים בוועדת הסל (N=70) אפשר להבחין בגורמים המרכזיים שתורמים ללגיטימציה ושמסכנים אותה. הערכת ההתאמה של אופן פעולת ועדת הסל לארבעת התנאים המפורטים במודל A4R (ציבוריות, רלוונטיות, ערעורים, ואכיפה) נעשתה על ידי ניתוח אותם מסמכי שיח ציבורי וראיונות חצי מובנים וכן מסמכים ממשלתיים המפרטים את נהלי עבודתה והערות שנכתבו בעקבות תצפיות משתתפות בפעילויות הקשורות לעבודת הועדה.

תוצאות:

ועדת הסל נהנית מתמיכה מתמשכת של הציבור הישראלי בלגיטימיות שלה. תמיכה זו נובעת בעיקרה מתפיסתה כמקצועית ושקופה. חזקות אלו של הועדה תואמות את הדגש על תנאי הציבוריות והרלוונטיות במודל ה A4R. שלושת גורמי הסיכון המרכזיים ללגיטימיות של ועדת הסל הם: (1) הרכב הועדה, (2) נהלי העבודה שלה, ו-(3) העקרונות המנחים שלאורה היא פועלת. חולשות אלו תואמות את הנקודות שבהן אופן פעולת ועדת הסל אינו הולם את מודל A4R. ממצאים אלו אפוא תומכים בתקפות האמפירית של מודל ה A4R.

מסקנה:

ניתוח ההתאמה בין עבודת ועדת הסל לבין מודל ה-A4R מצביע על עדכונים שיש לבצע בכל אבעת תנאי המודל על מנת שיכולתו להעריך לגיטימציה ציבורית תהיה מדויקת יותר. במקביל, ניתוח זה מדגיש את ההיבטים שבהם על ועדת הסל להשתפר על מנת להגביר את הלגיטימציה שלה, כגון שילוב ניתוח עלות-יעילות והכללת נציגי חולים בתהליך קבלת ההחלטות.

الخلفية:

يسرد قانون التأمين الصحي الحكومي، الذي سُن في عام 1995، قائمة الخدمات الصحية التي يحق لكل مقيم في إسرائيل الحصول عليها. كانت الحكومة تخصص ميزانية سنوية مخصصة لتحديث هذه القائمة على مدار العشرين عامًا الماضية. يتم تحديث القائمة وفقًا لتوصيات اللجنة العامة لتوسيع سلة الخدمات الصحية، المعروفة للجمهور باسم "لجنة السلة"، والتي تقوم بتقييم وتحديد أولويات جميع الأدوات التكنولوجية المرشحة. في هذا المقال، ندرس شرعية إجراء تخصيص الموارد هذا كما ينعكس في الخطاب العام في إسرائيل. نحن ندرس أيضًا مدى ملاءمة طريقة عمل لجنة السلة لمساءلة النموذج المحاسبي البيو أخلاقي accountability for reasonableness (باختصار، A4R).

الطرق:

يعتمد تقييم الشرعية الممنوحة للجنة السلة في الجمهور الإسرائيلي على تحليل وثائق الخطاب العام (مقالات من الصحافة المطبوعة، قرارات المحكمة، مناقشات في الكنيست، العدد = N=119). إن تحليل محتوى هذه الوثائق والمقابلات شبه المنظمة مع أصحاب المصلحة الرئيسيين في لجنة السلة (N = 70) يمكن للمرء أن يميز العوامل الرئيسية التي تسهم في الشرعية وتعرضها للخطر. تم إجراء تقييم مدى ملاءمة لجنة السلة للشروط الأربعة المنصوص عليها في نموذج A4R (الدعاية، والملاءمة، والطعون، والإنفاذ) من خلال تحليل وثائق الخطاب العام والمقابلات شبه المنظمة وكذلك الوثائق الحكومية التي توضح بالتفصيل عملها، والملاحظات، والتعليقات المكتوبة بعد الملاحظات التشاركية في الأنشطة المرتبطة بعمل اللجنة.

النتائج:

تتمتع لجنة السلة بدعم متواصل من الجمهور الإسرائيلي لشرعيتها. ينبع هذا الدعم بشكل أساسي من تصورها للمهنية والشفافية. تتوافق نقاط القوة هذه في اللجنة مع التركيز على الظروف العامة وأهميتها في نموذج A4R. إن عوامل الخطر الرئيسية الثلاثة لشرعية لجنة السلة هي: (1) تشكيل اللجنة، (2) وإجراءات عملها، و (3) المبادئ التوجيهية التي تعمل في ضوئها. تتوافق نقاط الضعف هذه مع النقاط التي لا يتناسب فيها سلوك لجنة السلة مع نموذج A4R. لذلك تدعم هذه النتائج الصلاحية التجريبية لنموذج A4R.

الاستنتاج:

يشير تحليل التطابق بين عمل لجنة السلة ونموذج A4R إلى التحديثات التي يجب إجراؤها على جميع الشروط الأربعة للنموذج حتى تكون قدرته على تقييم الشرعية العامة أكثر دقة. في الوقت نفسه، يسلط هذا التحليل الضوء على الجوانب التي تحتاج لجنة السلة إلى تحسينها من أجل زيادة شرعيتها، مثل دمج تحليل فعالية التكلفة وإشراك ممثلي المرضى في عملية صنع القرار.
